# Intuition and deliberation in elite expertise

**DOI:** 10.1186/s41235-026-00727-9

**Published:** 2026-04-21

**Authors:** Michael A. Vidulich, Pamela S. Tsang

**Affiliations:** 1 711th Human Performance Wing, U. S. Air Force Research Laboratory, Wright-Patterson Air Force Base, 2215 First Street, Building 33, Dayton, OH 45433–7028 USA; 2 Department of Psychology, Wright State University, Dayton, USA

**Keywords:** Decision making, Expertise, Time pressure, Chess performance

## Abstract

It has long been recognized that expert decision making entails both fast, intuitive and slower, deliberative processes. The enduring debate has to do with their relative roles. Some theories attribute the growth of expertise to the replacement of deliberative processes by intuitive perceptual recognition processes. Time pressure should have minimal effects on expert performance if intuitive processes are the primary basis for expertise. Two studies on archival data from the world’s strongest chess experts participating in high-stakes time-critical international matches with different time controls were conducted. Chess moves from 20 grandmasters and seven world chess champions were examined in Studies 1 and 2, respectively. Using a within-subject design, analysis of quantifiable performance measures in both speed (move time) and decision quality (blunder propensity) provided a strong demonstration of adverse time pressure effects. Experts did not deliberate only when time pressure was low. Importantly, elite chess players were highly strategic and adaptive in their deployment of time usage that allowed them to intuit when feasible and to deliberate when necessary. The present findings demonstrate the key role of deliberative processes even at the highest levels of expertise and are inconsistent with the assertion that intuitive processes are the primary basis for expertise. Discounting the deliberate component in expert decision making in theory and in practice could have far-reaching real-world consequences.

## Significance statement

Human decision making expertise is often the difference between safety and tragedy in emergencies. Pilots facing in-flight automation failures, surgeons at the operating table, or first responders at natural disaster sites are just a few examples of situations that demand rapid, effective expert decision making. So, understanding what expertise is and how it develops is vital. The present research demonstrates that a strong skill in deliberation plays a critical role in decision making at even the highest levels of expertise. When denied the opportunity to deliberate under time pressure, performance suffered. Expertise developed primarily through repeated exposures to the same decision episodes to hone the rapid recognition skill in order to facilitate fast intuitive decisions is unlikely to be, by itself, adequate in today’s increasingly high speed, dynamic operational environments. Deliberative processes will almost certainly be called upon during unprecedented or rarely practiced scenarios and they must be supported in real-world environments. Optimal real-world training must include building the necessary knowledge base to support effective deliberation when confronted by novel and unexpected event and the procedures to use that knowledge. Discounting the deliberate component in expert decision making in theory and in practice would have far-reaching real-world consequences.

## Introduction

Human decision making expertise is often the difference between safety and tragedy in emergencies. Pilots facing in-flight automation failures, first responders at natural disaster sites, or surgeons at operating tables are just a few examples of situations demanding rapid, effective expert decision making. So, understanding what expertise is and how it develops is vital. But there has been considerable controversy about the fundamental nature of decision making expertise. See for example, two recent publications each with a large collection of chapters on the topic: Ericsson et al. (2018) and Ward et al. (2019).

Early theories of expertise often sought to explain it as the development of automaticity. For example, Bryan and Harter (1899) found that telegraphic operators’ expertise grew in stages in which first the production of letters, then words, and then phrases became automated. Fitts and Posner (1967) developed this concept further with their three-stage theory of skill development: cognitive, associative, and autonomous stages. Prior to a skill becoming automated, performance would be guided by a slower and more conscious effort. Current theories of decision-making expertise have made pervasive use of dual process theories. Perhaps the most influential taxonomy has been Kahneman’s (2011) distinction between System 1 and System 2. Intuitive System 1 is fast, recognition-based, automatic, and relatively effortless; whereas System 2 is slower, controlled, and effortful. While it has long been recognized that both systems play a role in expert decision making, there is an enduring debate on their relative roles.

One popular and influential view in the applied literature is the Recognition-Primed Decision (RPD) model (Klein, 2015, 2021) that describes decision making expertise as primarily the acquisition of a repertoire of patterns through repeated exposures to the same or similar situations. A familiar situation, when encountered, would intuitively prime the appropriate decision. Although the RPD model incorporates both an intuitive pattern-recognition component and a deliberative mental simulation component, the intuitive component is clearly emphasized as the basis for expertise. As stated by Klein (2015, p. 165), “My own research suggests that people with experience rarely engage in the process of choosing among several options. Using their intuition, the patterns they have acquired, they usually identify an effective option as the first one they consider, based on the pattern recognition process”.

Training focused on the development of pattern-matching perceptual processes to support intuitive decision making typically emphasizes the repeated presentation of typical stimuli or scenarios in order to elicit the fast and correct response. This has become a popular training approach in many applied settings that include chess (e.g., Polgár, 1994; Smith & Tikkanan, 2018), military house-clearing operations (Harris-Thompson et al., 2006), nursing education (e.g., Nibbelink & Brewer, 2018), mental health (Ahuna & Becker, 2025), and sports (Bossard et al., 2022; Schweizer et al., 2011; Wang et al., 2024).

However, it has come to the attention of many that the tendency to discount the deliberate component in theory (e.g., Campitelli & Gobet, 2004) and in application (see Howell, 1997) could have widespread real-world significance. For example, Clewley and Nixon (2022) pointed out that despite the pilots of Asiana Airlines flight 214 being highly experienced with a combined 26,000 flight hours, they were unable to recognize that their aircraft was not within the approach safety criteria and therefore they did not perform the appropriate response protocol. This resulted in a number of fatal injuries and the aircraft destroyed. Clewey and Nixon posited that an overreliance on training to recognize typical events through simulation or operational flight experiences may leave pilots with inadequate event knowledge, and poorly positioned to recognize and respond to nontypical events. Similar concerns have been expressed in the training of airmen (e.g., Casner et al., 2013), firefighters (Lewandowsky et al., 1997), maritime search and rescue operators (Steigenberger et al., 2017), and surgeons (e.g., Moulton et al., 2010).

Chess often has been referred to as the drosophlia for studying human cognition (e.g., Bilalić et al., 2024; Charness, 1992; Simon & Chase, 1973). It has been used to study expertise for many reasons: (1) There exists a large pool of published competition data. (2) Elo ratings (Elo, 1978) provide a reliable objective index of expertise based on reproducible superior performance (Charness, 1992; Ericsson & Towne, 2010). On this scale, beginners would be rated below 1000 and grandmasters above 2500. (3) Competitive chess uses rigorous time controls. (4) Quantitative evaluation of the decision quality is available from powerful chess engines (e.g., Bilalić et al., 2024; Chabris & Hearst, 2003; Ericsson, 2014).

One approach to distinguish between intuitive and deliberative decision making processes is by studying the effects of time availability on decision quality. Experts are expected to be minimally affected under time pressure if their primary mode of decision making is based on quick intuition. By contrast, novices who need to rely on time-consuming deliberation due to insufficient experience would be adversely affected by time reduction for decision making.

Some studies of time constraints on expert chess performance have found minimal effects, supporting the intuition-based account of expertise. For example, Burns (2004) examined the game outcomes of blitz tournaments (5 min per game) and found that the rank scores of blitz game outcomes accounted for between 64 and 81% variance of the rank scores of their expertise ratings based on slower standard games. He concluded that skill differences were based largely on the effectiveness of fast intuitive processes. But one might quibble that a sizeable amount of variance (19–36%) would still need to be accounted for.

Calderwood et al. (1988) compared the performance of three class B players to three chess masters in a set of blitz games and a set of slower games (40 moves in 90 min). The weaker players performed poorer under time pressure (blitz games), whereas the masters seemed immune. It was concluded that deliberative processes must have played a minor role for the masters. However, in addition to the small sample size, this conclusion may have been undermined by the fact that the subjective move quality measure provided by a chess expert failed to distinguish between the two expertise groups in the nonblitz games even though their expertise ratings suggested that the masters should win almost 100% of the time (Burns, 2004). That is, the extent to which the effect of time pressure could be attributed to differences in chess expertise in this study is equivocal.

In another widely cited study, Gobet and Simon (1996, 2000) found that the performance of world champion Gary Kasparov was only slightly lower when playing simultaneous matches against multiple chess masters or grandmasters than his single matches. This was taken to support the notion that expert performance must be based primarily on fast intuitive processes. However, Montero (2019) noted that the drop in Kasparov’s Elo rating from 2750 to 2646 when playing the simultaneous matches was actually quite substantial. Van Harreveld et al. (2007) pointed out that such a drop would move Kasparov, then the strongest player in the world, to somewhere around 60th place. Montero and Van Harreveld considered the magnitude of the drop in performance to be too large to support Gobet and Simon’s conclusion that expert decision making was largely intuitive because it did not appear to be affected by time availability.

Other research has found more substantial effects of time constraints on expert chess performance. For example, Chabris and Hearst (2003) found that grandmasters (elite experts with Elo ratings from 2530 to 2790) made more and larger blunders in rapid games (15 min per game) compared to slower, standard games. Chang and Lane (2016) examined the distribution of times per move over the course of a game for weaker (Elo rating 1600–1699) and stronger (Elo rating 2300–2397) players. They found that even in blitz games, (a) chess players, especially stronger players, took considerable time on select moves, (b) stronger players made fewer blunders than weaker players, and (c) blunders made by stronger players tended to require deeper calculation than weaker players. This suggested that even experts deliberated at least some of the time in a fast moving blitz environment.

In a laboratory-controlled study, Moxley et al., (2012) had participants find the best move when presented with a tactical chess position while providing verbal protocols. Five minutes were given for each position. Move quality was evaluated objectively by computer software. Stronger players (mean Elo rating of 2194) selected better moves than the weaker players (mean Elo rating of 1836). Importantly, both weaker and stronger players benefited from additional time for deliberation. The move quality of the final moves chosen (after deliberation) were significantly stronger than those of first moves mentioned (with little opportunity for deliberation) in the verbal protocols, especially for the more difficult chess positions. If experts relied primarily on intuition akin to take-the-first heuristic (e.g., Raab & Johnson, 2007), the final move choice should be the same as the first move mentioned and there should be little difference between their move quality. Overall, the players chose the first move mentioned as their final move 45% of the time, which meant that the players often changed their mind. In fact, they were less likely to choose the first move mentioned as their final move choice for the more difficult positions (22% and 25% respectively for the stronger and weaker players) than for the easy positions (73% and 50% respectively for the stronger and weaker players). In other words, while intuitive first options worked reasonably well for the stronger players (73% of the time for the easy positions), they were more likely to deliberate for the difficult positions.

The present research further examines the roles of intuitive and deliberative processes in expert decision making as the debates continue in both theoretical developments and in applications (e.g., Brehmer, 1992; Cellier et al., 1997; Ericsson et al., 2018; Klein, 2015; Steigenberger et al., 2017; Ward et al., 2019). The research examines the effect of time pressure on performance as the brief review above suggests that performance under time pressure could be a distinguishing characteristic of the level of expertise. Time pressure should have minimal effects on expert performance if intuitive processes are the primary basis for expertise. Because both highly controlled laboratory studies and poorly structured real-world observations have their limitations in what they can inform, the present research examines a unique set of real-world elite chess performance in a natural, well-controlled, environment. Importantly, the level of experience was objectively ascertained by an international organization and quantitative measures that include both response times and decision quality were examined. And the decision quality was evaluated objectively by a powerful chess program that can be verified by others.

### Research approach

Two studies were conducted. Study 1 used an experimental approach similar to Calderwood et al. (1988) and Chabris and Hearst (2003) comparing performance in games with slower time controls to blitz games with stringent time controls. In addition, Study 1 tested and validated a new blunder propensity measure of decision quality. The new measure was evaluated not only in terms of its sensitivity to the different time controls, but also its sensitivity to the players’ game outcomes (i.e., win, lose, or draw).

Study 2 used the validated measure of decision quality to examine time pressure effects that emerged naturally within standard time control games based on the players’ time allocation decisions, not due to an overall time limit for the entire game. Time for each move from each player was examined to study the players’ strategic use of time throughout the game. This allows an independent examination of move time effects and the time constraint imposed by the total time available for the game. Study 2 was also unique in that all of the data were derived from World Chess Championship matches that pitted some of the top grandmasters in history against each other for the most important title in chess. This should provide a test of the upper limits of human expertise in the game of chess.

## Study 1 – effects of time pressure and measure validation

### Method

In this study, half of the games used a standard time control and half used a blitz time control for a within-subject comparison of time pressure effects. The standard time control allowed an average of 3 min per move and the blitz time control allowed an average of 7.5 s per move. Rather than trying to identify ‘true blunders’ of sufficient magnitude to potentially alter the course of a game (e.g., Chabris & Hearst, 2003; Chang & Lane, 2016) the present blunder propensity measure accumulated all computer identified blunders regardless of magnitude. This was designed to be sensitive to not only big blunders that might determine a game’s outcome, but also to the accumulation of smaller blunders that might degrade a player’s position until the game was lost. This new blunder propensity measure of decision quality will be validated against the actual game outcomes.

#### Data source and participants

The Dutch city of Wijk aan Zee hosts one of the most prestigious annual chess tournaments. In 1998 and 1999, a blitz chess tournament supplemented the standard time tournament (Geuzendam, 1998, 1999). Twenty players participated in both tournaments in at least one of the two years. Their Elo ratings ranged from 2540 to 2812 with a mean of 2671 (95% confidence interval (CI), [2639, 2704]). At the time of the tournaments, 8 of the grandmasters were ranked in the world top 10 according to the Fédération Internationale de Échecs (FIDE) (British Chess Magazine, ; Emms, 1999) and another 7 were rated in the top 64. The World Chess Champion, Garry Kasparov, competed in the 1999 tournaments.

#### Chess move evaluation

All games were analyzed with the Fritz 9.0 software (Fritz, 2005) in its Blundercheck mode that produced a numerical evaluation of the game position following each move. The score would be zero if the evaluation concluded that the game situation was even, positive if the calculation found an advantage for White, or negative if the evaluation indicated an advantage for Black. A score of 1.0 for White, or − 1.0 for Black, represented the equivalent of that player being about a pawn ahead in the game. This would be a considerable advantage.

In the present study, the absolute difference between the actual move played and the best move according to the Fritz evaluation was the blunder score, or the blunder size for that move. If Fritz listed no better alternative to the move played, the blunder score was zero. For analysis, blunder scores were summed and divided by the total number of moves to generate the Expected Blunder per Move (EBPM) representing the overall blunder propensity.

The Fritz software did not analyze every move of every game. The software ignored the moves from recognized chess openings as well as typical forced moves to a checkmate. The present analysis excluded all games that had fewer than five scored moves along with the games between the same players in the other tournament. For example, if the standard game for players X and Y was removed because of insufficient moves, then the blitz game between these two players was removed also. This ensured that the data for any player were based on the same opponents for the two tournaments and resulted in 104 pairs of games in the final dataset. This within-subject comparison enabled the most direct test of any impact of time pressure on performance (van Harreveld et al., 2007).

### Results

Data analysis focused on two issues: One, the players’ EBPM in the two tournaments (i.e., standard versus blitz) were compared to determine the impact of time pressure. Two, the players’ EBPM was compared to game outcomes to test its concurrent validity (Cronbach & Meehl, 1955).

First, Table 1 presents the means and 95% CIs for the two measures that are captured in EBPM—blunder size and proportion of blunders, for the standard and blitz tournaments. A two-tailed within-subject t-test showed that the mean proportion of blunders made (*t*_(19)_ = 6.54, Cohen’s *d* = 1.39) and the mean blunder size (*t*_(19)_ = 4.23, Cohen’s *d* = 0.96) were both statistically worse (*p* < .001) in the blitz than in the standard tournament with no overlap in the 95% CIs. Mean blunder size was about half a pawn larger in the blitz than in the standard tournaments. This difference is similar to that observed by Chabris and Hearst (2003) with participants at comparable skill level (mean Elo rating of 2666) as those in the present study. Although the standard condition was compared to a rapid game condition (about 1 min per move) in Chabris and Hearst’s study as opposed to the present blitz game condition, both sets of findings show a definite negative impact of reduced time on performance even for elite experts. The EBPM is examined more closely next.

**Table 1:** Blunders by tournament

	Tournament			
	Standard		Blitz	
Variable		95% CI		95% CI
Proportion of blunders	0.20	[0.18, 0.22]	0.27	[0.24, 0.29]
Mean blunder size	0.98	[0.80, 1.16]	1.45	[1.22, 1.68]
Mean EBPM	0.20	[0.15, 0.25]	0.38	[0.31, 0.45]

EBPM = Expected Blunder Per Move, CI = confidence interval

#### Expected blunder per move and time pressure

The EBPM scores took into account the blunder size and proportion of blunders. They were calculated for each player for all their moves in each tournament except for the moves not evaluated by the Fritz software. The blitz EBPM was higher than the standard EBPM for 18 out of 20 players. Table 1 shows that the mean blitz EBPM nearly doubled that of the standard EBPM. A two-tailed, within-subject *t*-test showed that the mean blitz EBPM was significantly higher than the mean standard EBPM (*t*(19) =  − 5.79, *p* < .001, Cohen’s *d* = 1.30) with no overlap in their CIs. The negative effect of time pressure on blunder propensity even for elite chess players was clear.

#### Expected blunder per move validation with game outcomes

Table 2 presents the Mean EBPM and 95% CIs for the standard and blitz tournaments subdivided by game outcome. For drawn games, the data for the White and Black players were analyzed seperately, but for decisive games, the data were combined regardless of player color for winners and losers. Naturally, the expectation was that losers of games would blunder more than winners. In drawn games, White and Black players would be expected to blunder equally. As expected, the winners’ EBPM was substantially lower than the losers’ with no overlap in their confidence intervals for either the standard or blitz tournament. In contrast, in drawn games the EBPM of White and Black players were very similar with substantial overlap of their confidence intervals. These results show that blunder propensity strongly influenced the game outcome.

**Table 2:** Mean EBPM by tournament and game outcome

	Tournament			
	Standard		Blitz	
Game outcome	EBPM	95% CI	EBPM	95% CI
Winner	0.15	[0.08, 0.21]	0.29	[0.24, 0.34]
Loser	0.39	[0.30, 0.48]	0.57	[0.49, 0.65]
White draw	0.13	[0.05, 0.20]	0.34	[0.25, 0.43]
Black draw	0.08	[0.07, 0.10]	0.25	[0.18, 0.32]

EBPM = Expected Blunder Per Move, CI = confidence interval

### Study 1 results summary

The results provided strong support for the validity of the EBPM measure—a single measure that captured both the blunder size and proportion of blunders made. The mean EBPM was strongly associated with the game outcome and similar findings were observed with different measures of chess performance from other studies. Importantly, time pressure clearly increased blunder propensity even for elite chess players, lending support for the importance of deliberative processes for optimizing chess performance.

## Study 2 – time pressure and blunder propensity in world championship matches

### Method

This study examined the game play of some of the greatest players in chess history in some of the most important games they ever played. Analyzing the time usage in these matches allowed examination of time pressure effects that emerged during the game rather than the overall impact of different time controls. Time per move and move quality were examined.

#### Data source and participants

The present dataset combined the games from 11 WCC (World Chess Championship) matches. A maximum of 24 games could be played in each match. A player earned one point for a won game, a half-point for a draw, and nothing for a loss. Accumulating 12.5 points would win the match. In the event of a 12–12 tie, the defending champion retained the title. In each game, 150 min were allotted for each player’s first 40 moves and an additional 60 min for each further set of 16 moves.

The dataset had seven competitors: Mikhail Botvinnik, Vladmir Smyslov, Mikhail Tal, Tigran Petrosian, Boris Spassky, Anatoly Karpov, and Garry Kasparov. All players were the defending World Champion in at least one of the matches in the dataset. Otherwise, they were the designated challenger for the title. These seven participants represent about a third of the population of world chess champions in the entire history of world chess championships.

#### Time per move

The 11 WCC matches studied were unusual in that time per move data were available in published reports (Clarke, 1969a, 1969b, 1969c; Golombek, 1954, 1957, 1958a, 1958b, 1958c, 1958d, 1961a, 1961b, 1961c; Kasparov et al., 1991; Keene & Goodman, 1985, 1986; Keene et al., 1987; Tal, 1970; Wade, 1964). These reports included each player’s cumulative time since the start of the game after each move, generally as integer minutes. When the reported times for the moves were more precise (less than one-half of one percent of the moves), the times were converted to the nearest tenth of a minute for the present analysis. Time per move was calculated by subtracting the cumulative time of the previous move from the cumulative time of the present move.

### Results

#### ***Time per move for moves 1 through ***45

**Fig. 1:**
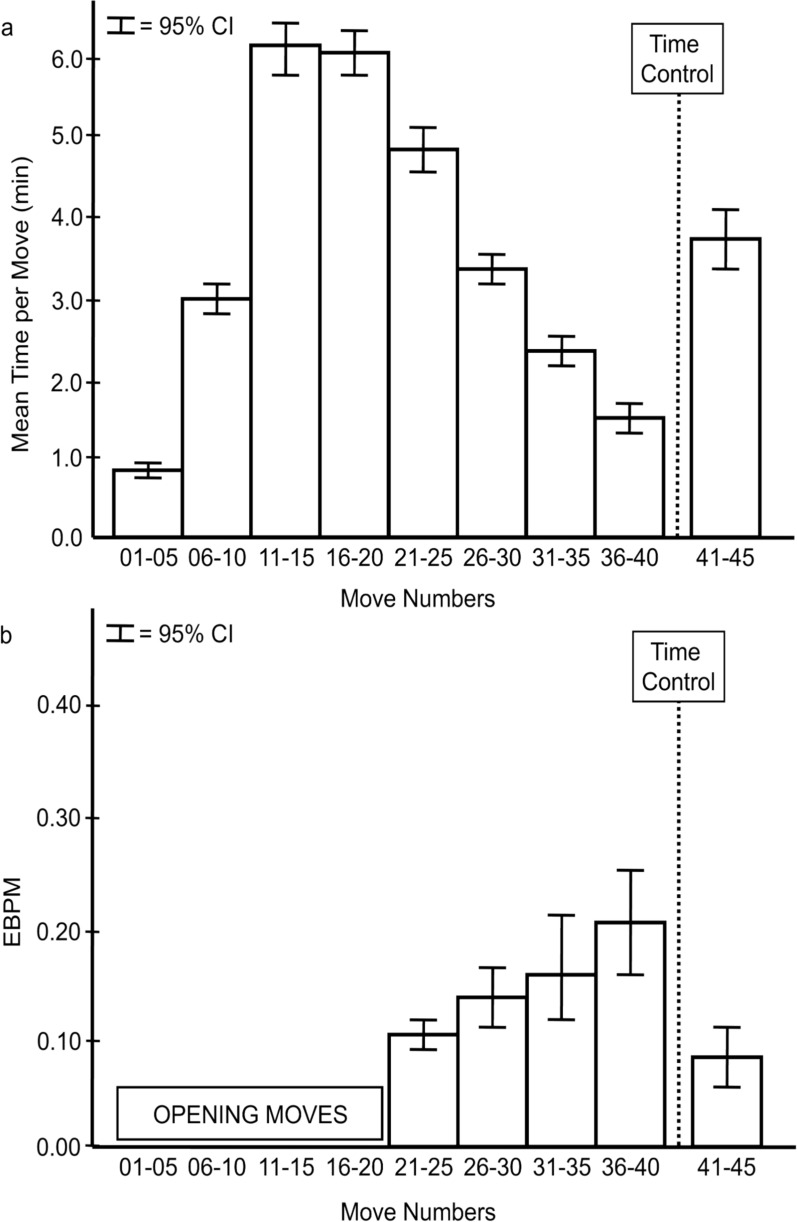
Time per move and Expected Blunder Per Move (EBPM) over the course of a game. **a** Time per move peaks around midgame and then becomes increasingly faster as time control is approached. **b** The EBPM increases as time control is approached beyond which, it is markedly reduced. Time Control marks the end of the first 40 moves that had to be completed within the allotment of 150 min

To examine time per move, the first 45 moves (the first 40 moves through the first time control of 150 min plus 5 moves past) were divided into groups of 5 moves. Although games could go beyond 45 moves, few reached the next time contol point of 56 moves. Figure 1a presents the means and 95% CIs for time per move of the first 45 moves.

As observed by Chang and Lane (2016) in faster blitz games, Fig. 1a shows that the time allocated by the players to each move varied dramatically over the course of a standard time-control game here as well. Opening moves (Moves 1–10) were made quickly. For Moves 11–20, the average time per move slowed to over 6 min. Then the average time quickened to less than 2 min by the last five moves just before the time control. However, the five moves immediately after time contol (Moves 41–45) slowed substantially. This pattern is highly similar to the results of Sigman and et al.’s (2010) who also found the opening and endgame to be played much faster than the middle game for even faster games with a time control setting of only 3 min per game. That is, chess players do not adopt a fast or a slow process exclusively. Considerable variation in time per move was observed across the game in our standard time control games, in Chang and Lang’s faster blitz games, and in Sigman et al.’s even faster games.

#### Expected blunder per move for moves 21 through 45

The Fritz chess analysis software did not evaluate any move that it identified as part of a stored sequence of typical opening moves. The first move that had Fritz evaluations as well as time data for all the moves was Move 21. So, the blunder data for Moves 21–45 were examined in the same fashion as the time per move. Whereas the time per move steadily decreased from Move 21 to Move 40 (Fig. 1a), the EBPM steadily increased (Fig. 1b). In contrast, immediately following time control, the substantial increase in time per move (Fig. 1a) was accompanied by a substantial reduction in EBPM (Fig. 1b) for Moves 41–45. That is, faster moves were associated with higher propensity for blunders, showing the importance of time for evaluating a move even for elite chess experts.

#### ***Microanalysis of the relationship between time per move and ***expected*** blunder per move***

While Fig. 1 displays the average move time within a band of move numbers, fast and slow moves within each band are examined here. Fast moves were defined as moves that were scored as using 0 min to accomplish. This meant that the person recording the cumulative times did not see enough movement of the clock to judge the precise amount of time used. Such fast moves were the moves least likely to allow any meaningful contribution from deliberative processes. Slower moves would have been more likely to allow deliberative processes to contribute to move selection.

**Proportion of Fast and Slow Moves.** Table 3 shows the proportion of fast and slow moves as a function of move number. There was a larger proportion of slow moves than fast moves in every band of move numbers except the first five moves. The higher proportion of fast moves at the beginning of a chess game is not surprising because chess games always start with the pieces in the same places. World class chess players would have studied many chess openings extensively. Interestingly, beyond the first five moves, the highest proportion of fast moves occured immediately before the time control point (Moves 36–40). So, the highest proportion of fast moves occured at the very beginning of the game when the pieces were in their most predictable places and for the five moves immediately prior to the time control point when there was the greatest chance of time pressure. Note that even for these presumably high time pressured moves, there were almost twice as many slow moves as fast moves.

**Table 3:** Proportion of fast and slow moves for moves 1 through 45

	Move NUMBERS								
Move type	1–5	6–10	11–15	16–20	21–25	26–30	31–35	36–40	41–45
Fast	.57	.31	.17	.13	.15	.21	.25	.34	.25
Slow	.43	.69	.83	.87	.85	.79	.75	.66	.75

The first 40 moves had to be completed prior to the 150-min time control. Fast moves took less than 1 min

**Fig. 2:**
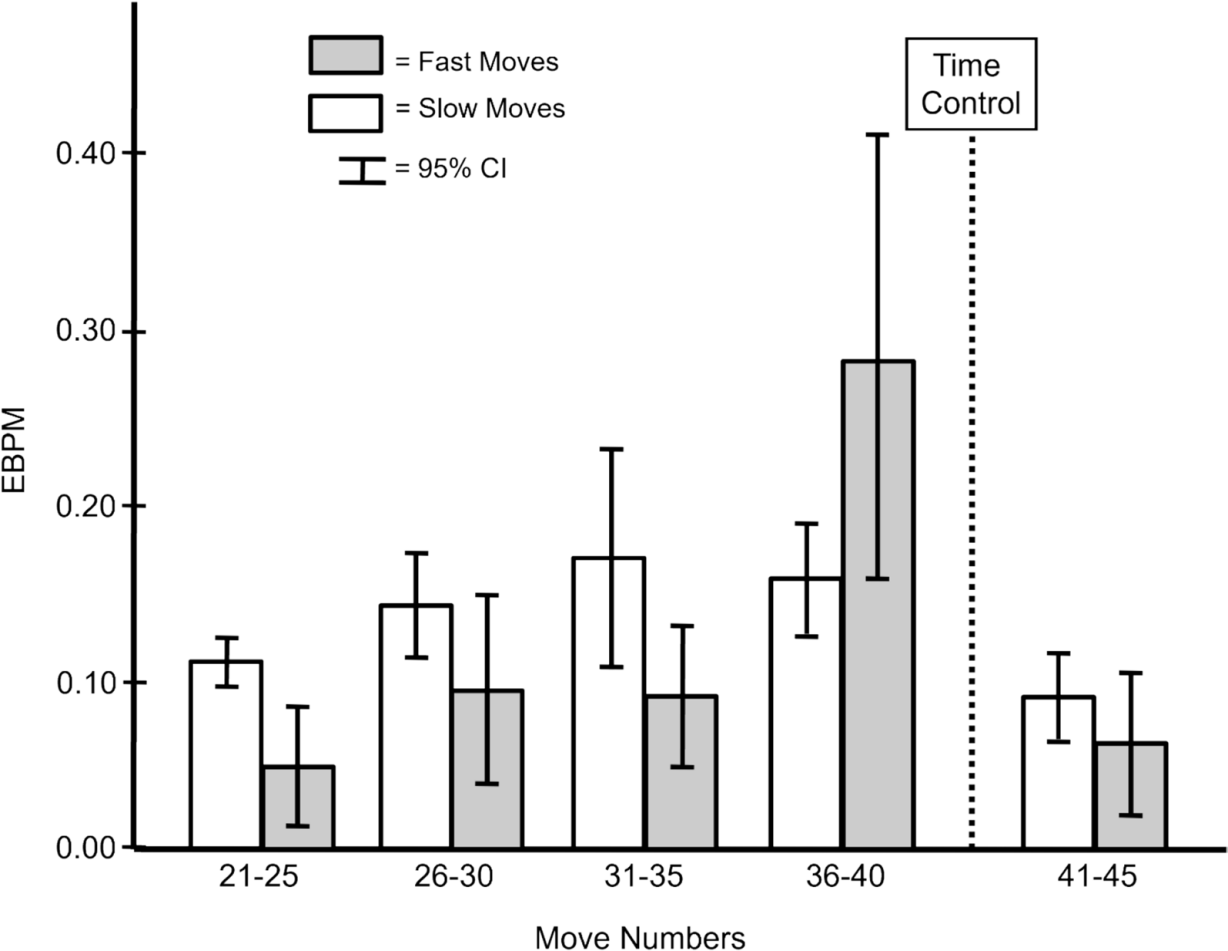
Expected Blunder per Move (EBPM) was lower for fast moves than slow moves except for moves immediately before time control when time pressure would be the greatest. Time Control marks the end of the first 40 moves that had to be completed within the allotment of 150 min

**Expected Blunder Per Move for Fast and Slow Moves.** Figure 2 shows the EBPM for the fast and slow moves from Move 21 to Move 45. Slow moves generally had higher EBPM than fast moves with one striking exception. Immediately before the time control (Moves 36–40), the proportion of fast moves increased (Table 3) and the EBPM spiked upward (Fig. 2). That is, although the fast moves in all of the other bands of moves were associated with smaller EBPM, when forced by an impending time control to move quickly, performance suffered a sharp rise in blunders.

**Fig. 3:**
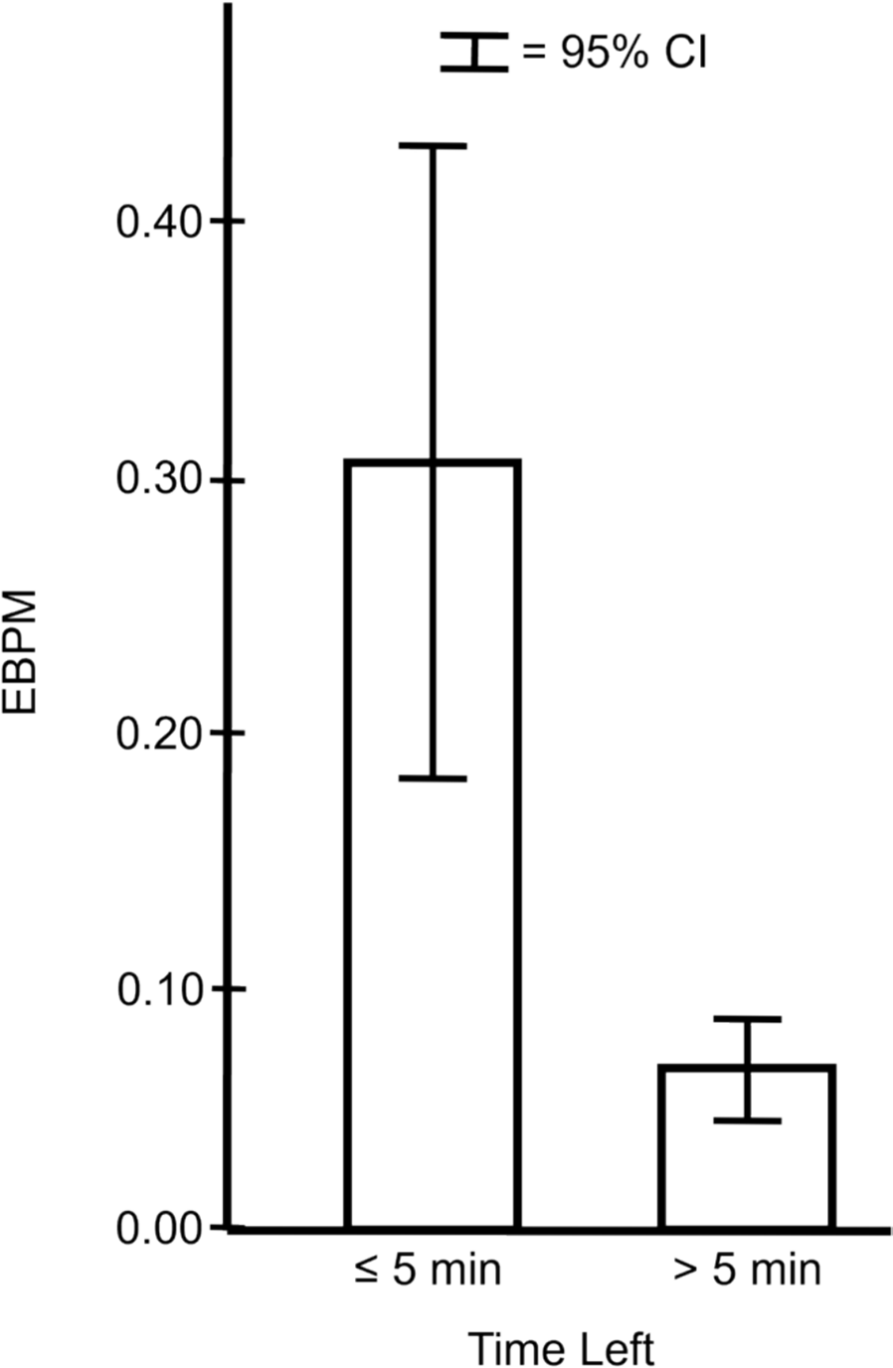
Fast moves under time pressure (≤ 5 min left) were associated with higher Expected Blunder Per Move (EBPM) than when not (> 5 min left)

**Time Pressure and Fast Moves Expected Blunder Per Move.** Figure 3 presents another view of the relationship between time constraints and fast move blunders. All fast moves from Move 21 and beyond were included in this analysis and separated into two categories: fast moves made with 5 min or less and fast moves made with more than 5 min remaining before the time control point, regardless of move numbers. Five minutes was selected as the cutoff point because Fig. 1a suggests that when ample time was available, the maximum mean time per move was approximately 6 min.

Figure 3 shows that not all fast moves were the same. The striking difference between fast moves made when there was more versus less than 5 min available shows that not all fast moves were necessarily indicative of expert’s infallible intuitive decisions. Time pressure could induce forced errors from elite chess players, even when there was a fairly large time window of five minutes (as opposed to seconds) remaining. Figures 2 and 3 show that the world chess champions were clearly able to identify situations that did not require deliberation even when the time available would have permitted deliberation. But when time pressure denied them any opportunity to deliberate, their performance suffered.

**Fig. 4:**
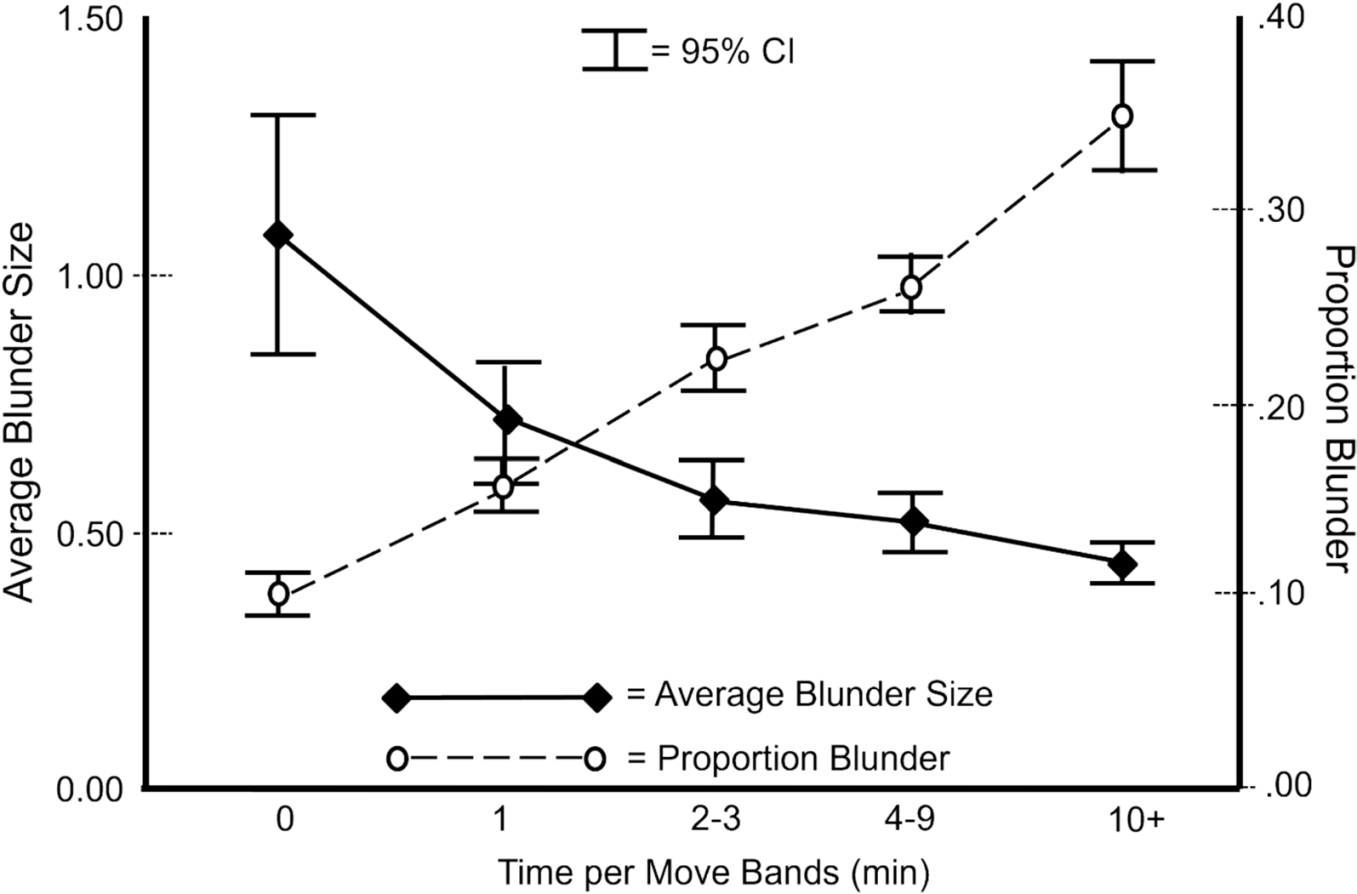
Fast moves (< 1 min) were associated with fewer and bigger blunders whereas slower moves were associated with more but smaller blunders. The number of moves included in the 0, 1, 2–3, 4–9, and 10 + bands were 2934, 2905, 2859, 2849, and 1250, respectively

**Proportion and Size of Blunders for Fast and Slow Moves.** In addition to time pressure effects, time per move also would be expected to depend on the difficulty or complexity of the chess position. One would expect that the more complex the position, the more time it would take to decide on the next move. The EBPM measure takes into account both the number of blunders made and the blunder size such that the same EBPM score could reflect a few large blunders or many small blunders. Figure 4 displays the two dimensions of EBPM at a finer time interval (as opposed to just fast vs. slow). The time per move data were grouped by the amount of time taken per move. Boundaries of the different time per move bands were set such that each band had approximately the same number of moves (datapoints) except for the 10 or more minutes band that had fewer moves. Time Band 0 included all the fast moves in Fig. 2; the rest were slow moves.

Figure 4 shows that fewest blunders were made among the fast moves (Time Band 0), but when blunders were made, they tended to be big blunders (more than 1 pawn) that could be consequential for game outcomes. Among the slow moves, the slower the moves, the more blunders were made but the smaller the blunder size. Judging by the proportion of blunders alone, one might conclude that slower deliberations were more blunder prone. But taking into account both the proportion and size of the blunders, the data suggested that the slower move time could be due to the difficulty of the chess positions. The increased move time might very well be what was needed to thwart bigger mistakes that would be more consequential to game outcomes.

### Study 2 results summary

Study 2 shows that the time pressure inherent in standard time control games exerted profound effects on elite chess performance. The time per move data revealed the highly variable decision times that emerged over the course of a game and the tradeoffs between the amount of time spent on a move and blunder propensity. As game circumstances forced a greater reliance on fast moves, blunder propensity increased. And not all fast moves were the same. Some were fast and accurate but those made under more time pressure were less so (Figs. 2 and 3). In fact, some fast moves were associated with big blunders (Fig. 4) that were likely to be consequential for game outcomes. These results show the importance of taking the decision quality into account when evaluating fast, presumably intuitive, moves and slow, presumably deliberative, moves.

## Discussion

The present results are a strong demonstration of time pressure effects with elite experts participating in high-stakes time-critical real-world events held in well-controlled settings. Lending further credence to the present findings are the within-subject design, objective assessment of expertise level, and quantifiable performance measures in both speed (move time) and decision quality (EBPM).

Consistent with the long-recognized notion that experts must make use of both intuitive recognition and deliberative search, both fast and accurate moves that were likely made intuitively as well as slower moves consistent with more deliberation were observed. However, the present findings are inconsistent with the assertion that fast intuitive processes are the primary basis for expertise (e.g., Burns, 2004; Calderwood et al., 1988; Chassy et al., 2023; Gobet & Simon, 1996; Klein, 2015). Study 1 provided empirical evidence for substantial negative effects of time pressure showing that performance of even experts of the highest skill level suffered when denied sufficient time to deliberate. Study 2 showed that a distinguishing skill of elite experts was their highly strategic and adaptive deployment of time usage that allowed them to intuit when feasible and deliberate when necessary. Theoretical and important practical implications of underestimating the deliberative component of decision making will be discussed.

### Effects of time pressure

Time pressure should have minimal effects on expert performance if intuitive processes are the primary basis of expertise. Study 1’s substantial negative effects of time pressure for even elite experts with difficult-to-match level of experience, adds to the findings from previous research that strongly point to the vital role deliberation plays in expert decision making. A common belief in the applied literature is that results from controlled laboratory research with inexperienced college students using a contrived task simply are not generalizable to real-world expertise (e.g., Klein, 2021; Klein et al., 2017). The studies highlighted in the present paper all used a naturalistic task that was either: (a) adapted for laboratory studies (Calderwood et al., 1988; Campitelli & Gobet, 2004; Moxley et al., 2012), or (b) used in actual real-world events. Among the real-world events sampled here were (a) chess moves and outcomes from huge databases from online chess servers (Burns, 2004; Chang & Lane, 2016; Russek et al., 2022; Sigman et al., 2010), and (b) chess moves and outcomes from chess tournaments (Gobet & Simon, 1996; van Harreveld et al., 2007; present Study 1), including world chess championship matches (Chabris & Hearst, 2003; van Harreveld et al., 2007; present Study 2). These studies together examined a range of skill levels from elite experts, lower level experts, as well as nonexperts. Results from laboratory-based studies and real-world events did not differ substantially. All of these studies show that intuition, by itself cannot account for the superb performance of experts.

That a strong skill in deliberation is an important component of expert performance was clearly demonstrated not just by the negative impact of time scarcity but also by the benefits of additional time on the decision quality. This was observed regardless of whether the time availability manipulation was done in a laboratory setting (e.g., Moxley et al., 2012) or by using different time controls (e.g., blitz versus standard time control) in a real-world setting (e.g., present studies, Chang & Lane, 2016; Charbris & Hearst, 2003; Russek et al., 2022; Sigman et al., 2010; van Harreveld et al., 2007). It should be noted that none of these studies equated deliberation with exhaustive search or comparison of all possible alternatives in parallel or in sequence. It is clear that no human chess player would be capable of such a feat, not even the strongest chess engine such as Stockfish 16 would be doing so. In fact, recent artificial intelligence research such as that of Schrittwieser et al. (2020) demonstrated that a combination of tree-search planning, akin to a deliberative process, and model-free reinforcement learning methods, akin to pattern-recognition-based intuition, was more successful than either approach alone in developing superior performance across a variety of games (i.e., chess, shogi, Go, Atari).

#### What if there is no time to deliberate?

The present data and other research showed that experts deliberated even with very little time (even with less than one minute). But what if there is not even a little time to deliberate? Much research has demonstrated that experts are also superior in their intuition in familiar situations. The present findings do not negate these findings. But there are also instances where there is not even time for intuition (that entails processes like pattern recognition) and one could only react by autonomic reflexes (as in swerving to avoid a deer). But autonomic reflexes are not conventionally thought of as a type of decision process.

### Experts’ adaptive use of time

So how do experts deliberate with so little time? As observed by Campitelli and Gobet (2004), experts can be highly strategic and adaptive in their deployment of time usage. This is evident in the elite players’ performance in the present Study 2. This finding also resonates with the findings from a growing body of chess studies (e.g., Chang & Lang, 2016; Russek et al., 2022; Sigman et al., 2010). Time per move varied considerably throughout the course of a game but with a typical pattern with fast familiar opening moves, slower midgame moves, and again fast moves when forced by the game’s time control. This was observed across a wide range of skill levels across studies and the move time variation increased with expertise (Chang & Lang, 2016; Sigman et al., 2010).

### Not all fast decisions are a result of infallible intuitions

The present findings showed that not all fast moves were the same. The WCC competitors were clearly able to intuitively identify opportunities to move quickly and accurately. But fast moves that were induced by impending time control were more blunder prone, likely because time pressure denied them the opportunity to deliberate. Whether intuition or deliberation is the primary basis for expert decision making cannot be distinguished by speed of deciding alone. The quality of the decision is as important an aspect as the speed of decision in understanding expert decision making.

### Limitations and generality

There are a number of limitations in our studies due to the archival source of the data. Here we suggest two lines of research to further study the interaction of intuitive and deliberative processes. One limitation of our present analysis was not having taken in account the opponent’s move time that the player could have used to consider their next move. Another limitation was not being able to use additional measures to help elucidate the role of intuition and deliberation in expert performance.

One limitation of the chess environment for studying the effects of time on decision making is that a chess player is not constrained to thinking about her move only after the opponent has made a move. To any degree possible, the chess player will no doubt be considering the possible moves the opponent might make and potential countermoves prior to the opponent actually making any move. If the player used the opponent’s move time to deliberate and correctly predicted the opponent’s move, the player’s fast and accurate move could appear to have been made intuitively, when it was actually the result of deliberation. But there is no way to ascertain if the expert player predicted correctly or intuited correctly based on the opponent’s move time. To examine any use of the opponent’s move time more closely, future research might have a human player compete with a computer chess engine set to respond at different intervals, including instant responses to the human’s moves. The instant response would eliminate any opportunity for the human to deliberate during the opponent’s move time. This would provide a more precise estimate of the amount of deliberation time, if any, that the human player used.

It is increasingly recognized that a combination of behavioral and neuroergonomic measures (Parasuraman, 2015) can help to better understand the underlying mental processes of complex tasks (Vidulich & Tsang, 2012, 2015). For example, global measures of mental workload and situation awareness (SA) could augment the information provided by the behavioral measures of decision time and decision quality used in the present research. Mental workload measures are meant to assess the degree to which a task is cognitively effortful. SA is the ability to perceive and comprehend the present environment and to project what is likely to occur in the near future (Endsley, 2018). An expert’s use of intuitive and/or deliberative processes to accomplish a task would be expected to have profound effects on their mental workload and SA. Intuitive processes should be associated with low mental workload. So, whether the expert was dealing with a simple, familiar task or a difficult task under time pressure, the mental workload impact of intuitive decision making should be low. If the expert’s deliberative processes are engaged to perform a task, the mental workload should be higher than in the intuitive case, and the degree of mental workload increase would be driven by the level of task difficulty. As for SA, in a simple familiar task the expert’s intuitive processes should reliably provide a high level of SA, but the SA would become less reliable as the task becomes more novel or complex. Bringing in deliberative processes to confront the challenges of novelty and complexity would result in slower response time and higher workload in an effort to construct better SA to support better decision making. This tradeoff is suggested by the data in Fig. 4 in which the fastest most intuitive moves were associated with the biggest blunders, presumably reflecting the impact of bad SA. And the slowest moves that presumably engaged the most deliberation showed the smallest average blunder size. This is consistent with the expert players effectively using their effortful deliberations to construct better SA for situations where their intuitions did not provide a quick and easy answer.

Ideally, in future studies, these complementary measures would be cognitively nonintrusive to not disrupt the ongoing decision making task and could provide continuous information about the cognitive processes involved throughout the game. Two promising candidates are the electroencephalogram (EEG) and the functional near-infrared spectroscopy (fNIRS) measures. The EEG records the brain’s electrical activity from electrodes attached to the participant’s scalp. Spectral power in several EEG frequency bands has been well-validated to reliably reflect the mental workload incurred in many task domains (e.g., Lei & Roetting, 2011; Puma et al., 2018) including chess (e.g., Williams et al., 2025). More recent studies have shown that it could also serve as a metric for SA (e.g., Festa et al., 2024; Zhang et al., 2023). The fNIRS provides a direct measure of cerebral oxygenation that increases with increased task demand. Although fNIRS technology does not have as long a history as that of the EEG, the fNIRS measure is being used to assess mental workload in an increasing number of domains (e.g., Ayaz et al., 2012; Sturman & Wiggins, 2021; Sturman et al., 2019) including chess (e.g., Leong et al., 2024). Recent studies have shown that the fNIRS measure could also serve as a SA metric (e.g., Festa et al., 2024).

The EEG and fNIRS measures are especially promising measures because they are well-validated measures in a number of domains and because of their relative portability that would be more amenable for in situ studies. However, as with many other physiological measures, the EEG and fNIRS measures do require specialized equipment and technical expertise to collect, analyze, and interpret the data.

The extent to which the present empirical demonstration of seamless strategic use of both intuition and deliberation by chess experts would apply to other domains will certainly require more work. However, similarities in a number of pertinent decision-making variables between chess and many other domains suggest that the present results would be relevant to any domain in which human expertise is needed for making rapid, effective decisions.

For example, Moulton et al. (2007) argued that the ability to transition between nonanalytic, automatic processes and effortful analytic processes as called for by the moment-to-moment situation is a hallmark of medical expertise. They pointed out that although it is the nonanalytic, automatic processes developed through extensive experience that allow experienced surgeons to perform standard operations with little effort while listening to music or conversing with the medical team, characterizing medical expertise exclusively by nonanalytic automatic processes is likely to be insufficient. This is because such processes would not be able to deal with novel or unusual situations such as when as anatomical anomaly was discovered unexpectedly during surgery. Here, a decision would likely have to be made in a time-pressured situation because once the first step was initiated, the subsequent steps would necessarily follow. A wrong decision could possibly allow the situation to devolve into a difficult-to-control state and pose considerable risk to the patient. In a subsequent empirical study, Moulton et al. (2010) documented that surgeons would slow down in the middle of a surgery when they came to a difficult part of a procedure or when they encountered an unexpected event. Their slowing down (or even stopping) was an indication of their transitioning into a more deliberate mode of operation. Activities that tend to accompany these slowdowns included removing distractions (such as turning off music or asking the medical team to stop talking) and even sweating—signs that they were engaged in more effortful tasks that demanded much attention. Highly experienced surgeons would purposely slow down to stay out of trouble, just like elite chess players would take more time to avoid large blunders.

As another example, playing chess and flying an airplane are seemingly disparate domains. But among their commonalities, time is of essence, possible courses of action are numerous, and uncertainty is involved whether one is interacting with humans or teams of machines. To wit, few would expect the 2009 landing on the Hudson River could be successful but for the high level of expertise of captain Sullenberger and co-pilot Skiles (with 19,663 and 15,643 total flight hours respectively). The time from the bird strike that caused a loss of engine power to the landing was less than four minutes. Both the real-time transcript and retrospective interviews showed that the captain had considered a number of options. The National Transportation Safety Board (NTSB, 2010) concluded that landing on the river provided the highest probability of survival. It should be noted that landing on a river due to bird strikes was not a common occurrence that experienced pilots would have repeated encounters with. As recounted by Captain Sullenberger, “we had never trained for this scenario. In our flight simulators, it was not possible to practice a water landing; the only training we got for a water landing was a theoretical classroom discussion and reading a few paragraphs in a manual”. (Hutchinson, 2019). In this case, Captain Sullenberger’s use of the very limited time available to deliberate effectively was essential to the successful landing.

### Implications for training

It is encouraging that a recent training effort was successful in changing the balance of deliberative and intuitive decision processes in experienced operational commanders in fire and rescue services (Cohen-Hatton & Honey, 2015). The effort was motivated by the observation that experienced commanders tended to initiate actions without making explicit plans potentially hindering the rescue effort (Cohen-Hatton et al., 2015). The researchers were concerned that while there are situations where quick intuitive decisions are useful, there could also be situations in which the action triggered by a cue might not be appropriate for the overall mission. Cohen-Hatton and Honey (2015) found that their goal-oriented training that incorporated the importance of evaluating goals, anticipated consequences, and risk/benefits analyses led to the commanders being more likely to show plan formulation before action execution. Importantly, these changes occurred without increasing the response latency to unexpected events.

An important implication from the present empirical work and review of the relevant literature is that accounts of expertise must not be satisfied with assuming that growing expertise is simply the displacement of slow deliberative processes by fast intuitive processes. Expertise developed primarily through repeated exposures to the same or similar decision episodes in order to hone the rapid perceptual recognition skill, as prescribed by the RPD model, is unlikely to be an adequate comprehensive training approach (e.g., Casner et al., 2013; Moulton et al., 2007; Steigenberger et al., 2017). Risks associated with an overreliance on such an approach includes the development of incompetence (i.e., bad habits, Yates, 2001), the development of inflexibility (Bilalić et al., 2008), and even recognition failure due to the inevitable variations in typicality in real-world events (Clewley & Nixon, 2022).

Because deliberative processes will almost certainly be called upon during unprecedented or rarely practiced emergencies in today’s increasingly high speed, uncertain operational environments, they must be supported in real-world environments. The expertise literature (e.g., Ericsson & Kintsch, 1995) argues that it is the experts’ vast, highly organized knowledge about principles and relationships that allow them to comprehend new situations amidst uncertainty and incomplete information and make rapid, effective decisions. An important implication for training is the need to impart trainees with more than surface-deep knowledge in order to also enable the development of their deliberative processes (see for example, Feltovich et al., 2018; Lane & Chang, 2018).

### Implications for theoretical developments

For theories of expertise, interactions of intuition and deliberation should be the focus and this points to the need for more such research as exemplified by the recent dual process theorizing work by Evans and Stanovich (2013), De Neys (2023), and Diederich and Trueblood (2018). A key finding of the present research is not just the importance of the role of deliberation in expert decision making. The present research suggests that a distinguishing skill of elite experts is their highly strategic and adaptive deployment of time usage that allow them to intuit when feasible and deliberate when necessary. Although this is not a new revelation (see for example, Brehmer, 1992; Cellier et al., 1997; Moulton et al., 2007; Feltovich et al., 2018; Steigenberger et al., 2017; Ward et al., 2018), the present findings underscore the need for more research on the flexibility that experts have in confronting unfamiliar, complex, dynamic environments, Theories of expertise and applications to support expert performance that minimize the role of deliberation in sustaining peak performance must be considered incomplete.

## Conclusions

The present research conducted an in-depth analysis of the decision making performance of elite chess players playing under varying degrees of time pressure. The results: (a) demonstrated unambiguously that a strong skill in deliberation is very much part of elite chess expertise in real-world competition and not just a trait of novices performing laboratory tasks, (b) demonstrated that the strategic and adaptive deployment of time use under time pressure is a hallmark of expertise, (c) demonstrated the importance of examining both decision time and decision quality for a better discrimination of intuitive and deliberative decision making, (d) established that the proposed decision quality measure, EBPM that captured both the size and propensity of errors, was a valid decision quality measure in the chess domain, and (e) argued that the distinction between intuitive and deliberative decision making has important theoretical implications for understanding expert decision making and practical implications for decision making training.

To improve real-world decisions, the present research suggests that more research will be needed: (a) to gauge the extent to which the present findings would apply to domains other than chess, (b) to understand the interactions of intuition and deliberation and the boundary conditions in which the different modes of decision making occur (see for example, Howell, 1997), and (c) to explore the most efficacious approach to training expert decision making because as Yates (2001) has pointed out, different modes of decision making demand different means for their improvement. Effective decision making is essential for maintaining efficiency and safety in complex environments so understanding and improving decision making performance must be a research priority.

## Data Availability

Data and analysis scripts for the studies are publicly available online at https://osf.io/9e3ng/.

## Abbreviations

CI: Confidence interval
EBPM: Expected Blunder per Move
EEG: Electroencephalogram
FIDE: Fédération Internationale de Échecs
fNIRS: Functional near-infrared spectroscopy
RPD: Recognition-Primed Decision
SA: Situation awareness
WCC: World chess championship

## Declarations

### Open practice statement

The chess game moves that were analyzed in the studies came from a database that accompanied the Fritz 9.0 software that was commercially available and are also available from online chess websites (e.g., chess.com or lichess.com). Study 2’s time per move data were available from published sources (see Reference List). Primary data and analysis scripts for both studies are publicly available at 10.17605/OSF.IO/9E3NG. The studies were not preregistered.
